# False negatives in *GBA1* sequencing due to polymerase dependent allelic imbalance

**DOI:** 10.1038/s41598-020-80564-y

**Published:** 2021-01-08

**Authors:** Jonas M. den Heijer, Arnoud Schmitz, Peter Lansbury, Valerie C. Cullen, Dana C. Hilt, Vincenzo Bonifati, Geert Jan Groeneveld

**Affiliations:** 1 Centre for Human Drug Research, Zernikedreef 8 2333 CL Leiden, The Netherlands; 2 Leiden University Medical Center, Leiden, The Netherlands; 3 GenomeScan B.V, Leiden, The Netherlands; 4 Lysosomal Therapeutics Inc, Cambridge, MA USA; 5 Department of Clinical Genetics, Erasmus MC, University Medical Center Rotterdam, Rotterdam, The Netherlands

**Keywords:** Clinical genetics, Genetic markers, Genotype, Medical genetics, Mutation, Sequencing, Genetics, Biomarkers, Neurology, Movement disorders, Neurodegenerative diseases, Parkinson's disease, Medical research, Genetics research, Clinical trial design

## Abstract

A variant in the *GBA1* gene is one of the most common genetic risk factors to develop Parkinson’s disease (PD). Here the serendipitous finding is reported of a polymerase dependent allelic imbalance when using next generation sequencing, potentially resulting in false-negative results when the allele frequency falls below the variant calling threshold (by default commonly at 30%). The full *GBA1* gene was sequenced using next generation sequencing on saliva derived DNA from PD patients. Four polymerase chain reaction conditions were varied in twelve samples, to investigate the effect on allelic imbalance: (1) the primers (n = 4); (2) the polymerase enzymes (n = 2); (3) the primer annealing temperature (T_a_) specified for the used polymerase; and (4) the amount of DNA input. Initially, 1295 samples were sequenced using Q5 High-Fidelity DNA Polymerase. 112 samples (8.6%) had an exonic variant and an additional 104 samples (8.0%) had an exonic variant that did not pass the variant frequency calling threshold of 30%. After changing the polymerase to TaKaRa LA Taq DNA Polymerase Hot-Start Version: RR042B, all samples had an allele frequency passing the calling threshold. Allele frequency was unaffected by a change in primer, annealing temperature or amount of DNA input. Sequencing of the *GBA1* gene using next generation sequencing might be susceptible to a polymerase specific allelic imbalance, which can result in a large amount of flase-negative results. This was resolved in our case by changing the polymerase. Regions displaying low variant calling frequencies in *GBA1* sequencing output in previous and future studies might warrant additional scrutiny.

## Introduction

Variants in the *GBA1* gene are, apart from the GWAS risk loci, the most common risk factor known to date to develop Parkinson’s disease (PD)^1,2^. Sequencing of the *GBA1* gene is known to be challenging, due to the highly homologous nearby pseudogene *GBAP1*^3,4^. *GBAP1* is not transcribed, but is in close proximity to *GBA1* and the exonic region of *GBAP1* shares 96% sequence homology with the coding region of the *GBA1* gene. False positive results are a well-known complication if highly homologous pseudogenes are not accounted for during sequencing. This can be overcome by using primers specific for the functional *GBA1* gene, long range amplification of the entire gene and by masking the pseudogene during alignment^4^.

We recently performed a large-scale screening of the *GBA1* gene in 3638 patients with Parkinson’s disease from the Netherlands, based on a next generation sequencing (NGS) protocol^5^. The pseudogene was accounted for by use of NGS with long-range polymerase chain reaction (PCR) and a primerset unique to the *GBA1* gene.

Here we report the serendipitous finding of an initially significant number of false negative results in our study, which could be readily solved by changing the polymerase enzyme. The corrected results were used for the previously published manuscript^5^. We noted that a *GBA1* variant that was previously detected in a patient in another study^6^, could not be confirmed by our sequencing method. Upon further investigation, the previous finding turned out to be a true positive result, while in our NGS method the variant was present, but it did not pass the default variant calling filter (heterozygous variant detected in more than 30% of the reads). A heterozygous allele should have a variant calling frequency of approximately 50% for both variants and a homozygous allele should have a variant calling frequency of approximately 100%, with very little noise using modern techniques^7^. Upon experimental lowering of this variant calling filter to 2%, the total *GBA1* variant hit-rate almost doubled, primarily driven by the relatively common NM_000157.3:c.1093G > A;p.(Glu365Lys) (allelic name E326K) variant.

This paper describes how changing the polymerase enzyme normalized all variant frequencies, thereby uncovering the false negative results, by using a structured assessment of different primers, PCR primer annealing temperatures (T_a_), amounts of DNA input and two different polymerases.

## Results

### Genotyping

**Figure 1:**
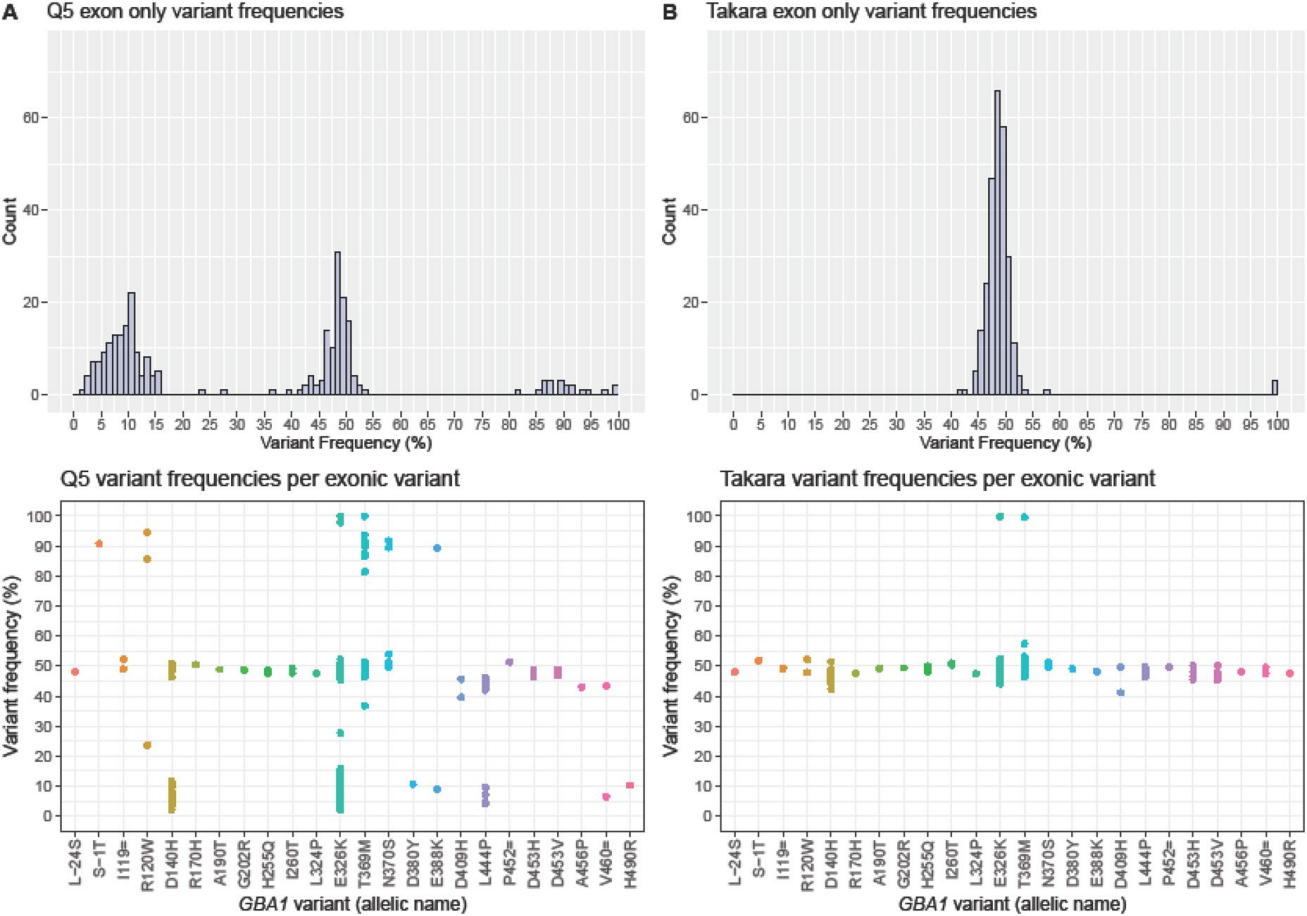
Comparison of exonic variant frequencies using (**A**) Q5 polymerase or using (**B**) TaKaRa polymerase. These are the first 216 samples with a suspected GBA1 exonic variant, initially sequenced using the Q5 polymerase and later repeated with TaKaRa polymerase. The top row shows histograms combining all exonic variants. The bottom row shows dot plots with variant frequencies per specific exonic variant. In normal circumstances, one would only expect a variant frequency around 50% and 100%. 44 samples contained two exonic variants (primarly p.[Asp179His;Glu365Lys] (D140H + E326K)), one sample contained three variants and one sample four. Frequencies below 30% are generally filtered out; the filter was customly set to 2% here. Variant details can be found in our previous publication^5^.

Initially, 1295 samples were sequenced using Q5 High-Fidelity DNA Polymerase. 112 samples (8.6%) had an exonic variant with a variant frequency higher than 30%. An additional 104 samples (8.0%) had an exonic variant with a variant frequency lower than 30%, see Fig. 1A.

The pattern of normal and abnormal variant frequencies is depicted per exonic variant in Fig. 1A,B, bottom row). Some variants were only detected at a normal or abnormally low read frequency (e.g. c.1093G > A;p.(Glu365Lys) (E326K) and NM_000157.3:c.1448 T > C;p.(Leu483Pro) (L444P)), some variants were only detected normally or abnormally high (e.g. NM_000157.3:c.1223C > T;p.(Thr408Met) (T369M) and NM_000157.3:c.1226A > G;p.(Asn409Ser) (N370S)) and some variants could be either low, normal or high. If a sample had multiple variants, the imbalance was consistent over variants.

Based on intronic variants, including known benign variants, many samples without exonic variants were also imbalanced. Due to uncertainty of sequencing in GC-rich and repeat regions, some intronic variants with a low frequency could be sequencing or mapping errors as opposed to imbalanced amplification. Therefore, allelic balance could not be assessed for all samples without an exonic variant (data not shown). An overview of all intronic and exonic variant frequencies of all samples sequenced by Q5 polymerase is given in Supplementary Fig. 1.

### Assessment of PCR conditions

**Figure 2:**
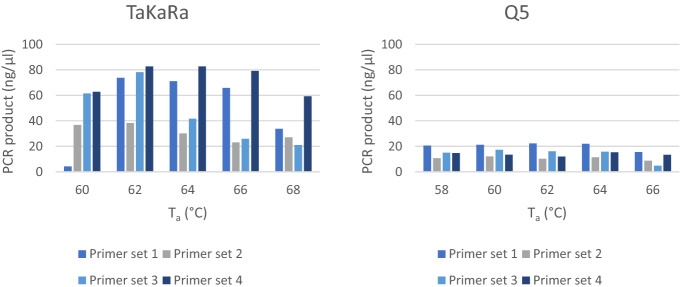
Varying T_a_ and the respective amount of PCR product, using commercial human DNA (100 ng) with four different primer sets, split for the TaKaRa and Q5 polymerase. T_a_ = primer annealing temperature; PCR = polymerase chain reaction.

PCR yield of human control DNA per T_a_ for Q5 and TaKaRa using all four primer sets can be seen in Fig. 2. PCR yield using TaKaRa was generally higher than using Q5. A T_a_ of 62 °C for Q5 and 63 °C for TaKaRa was chosen.

The PCR product increased with increasing DNA input (4 ng, 20 ng, 100 ng) of the human control samples. Using the PD samples, primer set 2 had the lowest yield. Samples using primer set 2, control samples with 4 ng DNA input and negative controls were omitted from library preparation and sequencing. Variant frequencies per polymerase and per primer set of control samples with varying DNA input can be seen in Table 1 and variant frequencies of the PD samples can be seen in Table 2. Difference in DNA input did not affect the variant frequencies, based on control samples with 20 ng or 100 ng input. Choice of primers did not affect the variant frequencies, based on three different primer sets unique to the *GBA1* gene. Samples amplified using TaKaRa polymerase showed balanced variant frequencies, including the initially imbalanced samples using Q5 polymerase. PD sample 1 had a low yield after PCR using primer set 1 and TaKaRa polymerase and PD sample 9 had a low yield after PCR using primer set 3 and Q5 polymerase, therefore these two samples could not reliably be analyzed.

**Table 1:** Variant frequencies based on DNA input of 20 ng and 100 ng of commercial human control samples.

	Benign SNP	TaKaRa			Q5		
		Primer 1	Primer 3	Primer4	Primer 1	Primer 3	Primer4
Control 100 ng	rs762488	49.1	48.2	47.2	33.5	32.1	28.1
Control 20 ng	rs762488	46.3	52.2	44.4	35.7	32.8	30.1
Control 100 ng	rs2075569	49.8	50.2	48.8	32.1	30.9	27.3
Control 20 ng	rs2075569	47.8	47.9	46.2	33.9	31.9	28.7

4 ng was not sequenced due to a low yield.

**Table 2:** Variant frequencies of GBA-PD samples based on three different primer sets unique for the functional GBA1 gene and two different polymerase enzymes.

Sample	Variant	TaKaRa			Q5		
		Primer 1	Primer 3	Primer4	Primer 1	Primer 3	Primer4
1	E326K	Low yield*	99.9	99.8	100	100	100
2	E326K	99.8	99.9	99.8	99.9	100	100
3	E326K	51.2	53.2	51.7	47.7	48.3	50.4
4	E326K	48.6	50.4	50.5	47.5	49.4	51.4
5	E326K	48.1	47.6	47.1	33.4	32.3	31
6	T369M; L324P	50.7; 47.1	48.1; 50.3	50.7; 47.8	50; 47.7	49.5; 49	50.8; 48
7	T369M	48.7	50.8	49.7	63.3	64.5	68.3
8	L444P	44.4	42.4	43.9	27.8	28.3	23.6
9	E326K	48.4	49.8	48.4	34.2	Low yield*	29.8
10	E326K	49	47	48.7	27	28.6	23.6
11	E326K	48.5	48.3	49.9	24.8	23	18.8
12	N370S	49.5	53.1	53.7	51.5	53.5	52.9

See Table 3 for HGVS nomenclature.

*Two samples had a relatively low yield after PCR and could not reliably be sequenced.

### Confirmation of genotype

All samples with an exonic *GBA1* variant based on Q5 polymerase (variant frequency ranges: 2.0–23.7% (n = 58), 39.6–57.4% (n = 144), 81.4–94.5% (n = 11) and 99.8–100% (n = 3)), were confirmed using TaKaRa polymerase (variant frequency ranges: 44.8–57.4% (n = 213) and 99.4–99.8% (n = 3)), see Fig. 1A,B. All samples with a variant frequency between 80 and 95% using Q5 polymerase turned out to be heterozygous using TaKaRa polymerase, so in these samples there was an allelic imbalance in favor of the allele containing the *GBA1* variant over the reference while using Q5 polymerase.

## Discussion

This paper describes the serendipitous finding of a polymerase dependent allelic imbalance when sequencing the GBA1 gene, resulting in a high number of false negative results. In our cohort, the variant hit rate increased by 93% after changing the polymerase, primarily driven by the relatively common c.1093G > A;p.(Glu365Lys) (E326K) variant and the [c.535G > C];[c.1093G > A];p.[(Asp179His);(Glu365Lys)] (D140H + E326K) complex allele (a likely Dutch founder allele). This artifact was initially disguised by the commonly used variant calling filter set at a frequency of 30%. Our previous publication^5^ was based on correct data after this artifact was detected and corrected. Considering this finding, we strongly advise to further explore the lower frequency regions of GBA1 sequencing output in previous and future cohorts. Similar allelic imbalance in sequencing studies can potentially have a major impact on the prevalence of GBA1 variants reported in other populations.

Preferential engagement of the polymerase to a specific allele can have multiple causes, like differences in GC content between alleles, heterozygous variants in the primer region, methylation status or altered folding^8–11^. A different variant in the primer annealing site was excluded as a cause due to equivalent results when using different primer sets. No specific intron or exon variant could be detected that differentiated between balanced and imbalanced samples. At this point, it remains unclear what causes this imbalance. Similarly, it is unclear how this translates to other polymerases or whether this could be resolved using modified PCR conditions. Concentration of the Q5 polymerase enzyme could not be varied, because this is provided in a mastermix solution.

Methylation status can alter the DNA secondary structure and melting properties^10^. Amplification is not prevented by methylation, but it is rather driven preferentially toward the unmethylated allele when the methylation status of the two alleles is distinct^11^. Similarly, altered DNA strand folding, like hairpin and G-Quadruplex structures, can also interfere with PCR^12,13^. Coadjuvants to improve DNA denaturation, and performing PCR amplification in a KCl free buffer have been suggested to circumvent these problems^11,12^. Histone modifications are known to be involved in epigenetic modification^9^, but these are typically removed during DNA purification, so an effect on PCR seems unlikely.

A previous assessment of allele dropout showed a majority to be caused by nonreproducible PCR failures rather than sequence variants^14^, but this seems unlikely in the current report, considering the widespread and reproducible imbalances reported.

Most exonic variants, if abnormal in read frequency, displayed a decrease in frequency to below 30%. Some exonic variants however, like c.1223C > T;p.(Thr408Met) (T369M) and c.1226A > G;p.(Asn409Ser) (N370S), only showed normal or abnormally high read frequencies. Samples with a variant frequency up to 94.5% turned out to be heterozygous (correct frequency ~ 50%) after changing to TaKaRa polymerase. This shows that also marginally abnormal results should be interpreted with caution.

Most exonic variants that occurred in an imbalanced frequency in certain samples, were also seen in a normal frequency in other samples (using Q5 polymerase). This implies that the exonic variant is not exclusively responsible for the imbalance. Conversely, exonic variants that were only seen in a normal frequency, are not precluded from a potential imbalance, because these variants were less prevalent and may therefore have only been detected with balanced frequencies by chance. Using the TaKaRa polymerase, the allelic imbalance was eliminated for all exonic variants and most intronic variants, but the imbalance could still be seen for some remaining intronic variants (Supplementary Figs. 2 and 3). These intronic variants mostly were in high-repeat intronic regions, or in other regions with a relatively low coverage, considered technical noise. In samples containing these imbalanced intronic variants, other intronic (and sometimes exonic) variants did have balanced frequencies, strengthening the notion that the imbalanced intronic variants were a technical artifact.

These findings could also explain discrepancies between multiple reports from the same or nearby populations. Both in the United Kingdom and in Spain, the c.1093G > A;p.(Glu365Lys) (E326K) variant was initially not found^15,16^, but in later cohorts it was reported^17–19^.

Considering the ongoing drug development targeting the *GBA1* pathway, more and more people with Parkinson’s disease will be screened for *GBA1* variants. Both for counseling purposes and for adequate enrollment in upcoming clinical trials, a reliable sequencing method is essential.

## Material and methods

### Participants

PD patients were included in the Netherlands as described previously^5^. This study was approved by an Independent Ethics Committee of the Foundation ‘Evaluation of Ethics in Biomedical Research’ (Stichting Beoordeling Ethiek Biomedisch Onderzoek), Assen, The Netherlands. Written informed consent was obtained from all participants according to the Declaration of Helsinki.

### Genotyping

Saliva was obtained from patients using Oragene DNA OG-500 tubes (DNA Genotek). DNA isolation, next generation sequencing (NGS) and data analysis was performed by GenomeScan B.V., Leiden, the Netherlands, as described previously^5^. The *GBA1* gene was unambiguously amplified using primers unique to the functional gene; these primers were used previously^20^. Initially, the Q5 Hot Start High-Fidelity 2X Master Mix (New England BioLabs Inc.) was used. After the imbalanced variant frequencies were detected, a structured assessment of various PCR conditions was conducted.

DNA was isolated according to standardized procedures using the QIAsymphony DSP DNA Midi Kit (Qiagen). The DNA concentration of the samples was determined using Picogreen (Invitrogen) measurement prior to amplification. Afte PCR, the long-range PCR product was fragmented using the Bioruptor Pico (Diagenode) to an average size of 300–500 bp before sequencing on an Illumina sequencer. Library preparation was performed using the NEBNext Ultra II DNA Library Prep kit (New England Biolabs E7370S/L). End repair/A-tailing, ligation of sequencing adapters and PCR amplification was performed according to the procedure described in the NEBNext Ultra DNA Library Prep kit instruction manual. The quality and yield of the library preparation was determined by Fragment Analyzer analysis. Clustering and DNA sequencing (paired-end 150 bp) using the Illumina cBot and HiSeq 4000 was performed according to the manufacturer's protocols. Image analysis, base calling and quality check was performed with the Illumina data analysis pipeline RTA v2.7.7 and Bcl2fastq v2.17.

Data analysis was performed using a standardized in-house pipeline developed by GenomeScan B.V., based on the Genome Analysis Toolkit’s (GATK) best practice recommendations^21^, including instructions for raw data quality control, adapter trimming, quality filtering, alignment of short reads, and frequency calculation. During the alignment step (using Burrows-Wheeler Aligner v0.7.4) to the human reference (hg19), the *GBAP1* gene was masked due to the high homology with *GBA1*. By masking *GBAP1*, mapping quality of *GBA1* reads increased, especially at the 3′-prime of the gene, where the homology is the highest.

*GBA1* variants are described based on the amino acid position excluding the 39-residue signal sequence at the start (also known as “allelic nomenclature”), which is used historically in *GBA1* research (format: E326K). The recommended nomenclature by the Human Genome Variation Society (HGVS) is also given (format: p.(Glu365Lys)) and further variant details can be found in our previous publication^5^. The used NCBI Reference Sequence is NM_000157.3, NP_000148.2, assembly GRCh37.

### Assessment of PCR conditions

To investigate the cause of the variant frequency imbalance, four PCR conditions were varied: (1) the primers (n = 4); (2) the polymerase enzymes (n = 2); (3) the primer annealing temperature (T_a_) specified for the used polymerase; and (4) the amount of DNA input.

Twelve samples with a *GBA1* variant of varying variant frequencies were further analyzed for this purpose: two homozygous samples, four heterozygous samples (with balanced variant frequencies) and six samples with an abnormal variant frequency, see Table 3. Additionally, commercial human DNA and negative controls were used.

**Table 3:** Selection of twelve PD samples with varying GBA1 variant frequencies.

Sample	*GBA1* (HGVS)	*GBA1* (allelic name)	Variant frequency
1	p.(Glu365Lys)	E326K	100
2	p.(Glu365Lys)	E326K	97.8
3	p.(Glu365Lys)	E326K	47.4
4	p.(Glu365Lys)	E326K	50.9
5	p.(Glu365Lys)	E326K	9.3
6	p.([Leu363Pro];[Thr408Met])	T369M; L324P	50.5; 47.5
7	p.(Thr408Met)	T369M	87.4
8	p.(Leu483Pro)	L444P	9.6
9	p.(Glu365Lys)	E326K	10.6
10	p.(Glu365Lys)	E326K	5.2
11	p.(Glu365Lys)	E326K	3.7
12	p.(Asn409Ser)	N370S	50.9

Samples 5, 8, 9, 10 and 11 have an abnormally low variant frequency and sample 7 has a frequency too high for a normal heterozygous and too low for a normal homozygous variant. Sample 6 is compound heterozygous (T369M on one allele and L324P on the other allele). Both the HGVS nomenclature is given and the GBA1 allelic name, which excludes the 39-amino acid signaling peptide, both using accession NP_000148.2

The four primers investigated can be found in Table 4. Primer set 1 was used throughout the original genotyping project. The two polymerases used were Q5 Hot Start High-Fidelity 2X Master Mix (New England BioLabs Inc.) and TaKaRa LA Taq DNA Polymerase Hot-Start Version: RR042B. Q5 DNA Polymerase is composed of a novel polymerase that is fused to the processivity-enhancing Sso7d DNA binding domain^22^. TaKaRa LA Taq DNA Polymerase combines Taq DNA polymerase and a DNA-proofreading polymerase, with 3′ → 5′ exonuclease activity^23^.

**Table 4:** Four different GBA1 primer sets that will lead to amplification of the functional gene and not the pseudogene GBAP1.

*GBA1* primer set	Name	Sequence (5′- > 3′)	Start genomic position
1 Mata et al.^20^	GBA_P1_F	GTTGTCACCCATACATGCCC	chr1: 155204150
	GBA_P1_R	CTCTCATGCATTCCAGAGGC	chr1: 155211199
	Product length	7050	–
2 Jeong et al.^24^	GBA_P2_F	TCCTAAAGTTGTCACCCATACATG	chr1: 155202292
	GBA_P2_R	CCAACCTTTCTTCCTTCTTCTCAA	chr1: 155211206
	Product length	8915	–
3 Liu et al.^18^	GBA_P3_F	CGACTTTACAAACCTCCCTG	chr1: 155203972
	GBA_P3_R	CCAGATCCTATCTGTGCTGG	chr1: 155211737
	Product length	7766	–
4 Designed by GenomeScan using NCBI primer blast	GBA_P4_F	AGAAGTTGATCCCGGTGCTG	chr1: 155202617
	GBA_P4_R	ATGATGAAACAAGGGACGCTGC	chr1: 155211676
	Product length	9060	–

Genomic position based on Hg19.

First, the optimal primer annealing temperature (T_a_) was assessed for both polymerases, using all four primer sets and 100 ng commercial human DNA. A standard three-step PCR cycle was performed according to the instructions of the supplier. For the Q5 polymerase, a T_a_ of 58–66 °C with increments of 2 °C was investigated and for the TaKaRa polymerase, a T_a_ of 60–68 °C with increments of 2 °C was investigated. Concentrations of the PCR products were measured using Picogreen (Invitrogen). The size of the PCR products was determined by Fragment Analyzer analysis.

Using the determined T_a_ per polymerase, the four primers were assessed using the twelve DNA samples from PD patients, commercial human control DNA and a No Template Control. The commercial human DNA was used to vary the DNA input, using 4 ng, 20 ng and 100 ng. For all PD samples, 100 ng was used. See Table 5 for an overview.

**Table 5:** Analysis setup to investigate the effect of four primers sets and of DNA input on variant frequencies. This setup was performed once using Q5 polymerase and once using TaKaRa polymerase.

DNA input (ng)	Primer set 1	Primer set 2	Primer set 3	Primer set 4
100	Sample1	Sample1	Sample1	Sample1
100	Sample2	Sample2	Sample2	Sample2
100	Sample3	Sample3	Sample3	Sample3
100	Sample4	Sample4	Sample4	Sample4
100	Sample5	Sample5	Sample5	Sample5
100	Sample6	Sample6	Sample6	Sample6
100	Sample7	Sample7	Sample7	Sample7
100	Sample8	Sample8	Sample8	Sample8
100	Sample9	Sample9	Sample9	Sample9
100	Sample10	Sample10	Sample10	Sample10
100	Sample11	Sample11	Sample11	Sample11
100	Sample12	Sample12	Sample12	Sample12
100	Human control	Human control	Human control	Human control
20	Human control	Human control	Human control	Human control
4	Human control	Human control	Human control	Human control
0	Negative control	Negative control	Negative control	Negative control

Samples one to twelve were previously assessed to have a GBA1 variant using Q5 polymerase, some with abnormal variant frequencies, see Table 3. Negative control samples contained no DNA.

### Confirmation of genotyping

All samples with a *GBA1* exonic variant, either balanced or imbalanced, were rerun in the new conditions based on the above mentioned assessment.

## Supplementary Information

Supplementary Figures.

## Data Availability

The data that support the findings of this study are available from the corresponding author upon reasonable request.

## Abbreviations

GATK: Genome analysis toolkit
HGVS: Human Genome Variation Society
NGS: Next generation sequencing
PCR: Polymerase chain reaction
PD: Parkinson’s disease
T_a_: Primer annealing temperature
