# Supplementary material for: False negatives in GBA1 sequencing due to polymerase dependent allelic imbalance
Source: Sci Rep. 2021 Jan 8;11:161. doi: 10.1038/s41598-020-80564-y (PMC7794395; doi:10.1038/s41598-020-80564-y)
Supplement: Supplementary file 1 — Supplementary Figures. [file 41598_2020_80564_MOESM1_ESM.docx]

False negatives in *GBA1* sequencing due to polymerase dependent allelic imbalance

Jonas M. den Heijer, MD, 0000-0002-2272-7978 ^1,2^, Arnoud Schmitz, MSc^3^, Peter Lansbury, PhD^4^, Valerie C. Cullen, PhD^4^, Dana C. Hilt, MD^4^, Vincenzo Bonifati, MD, PhD^5^, Geert Jan Groeneveld, MD, PhD^1,2^

^1^Centre for Human Drug Research, Leiden, The Netherlands

^2^Leiden University Medical Center, Leiden, The Netherlands

^3^GenomeScan B.V., Leiden, The Netherlands

^4^Lysosomal Therapeutics Inc, Cambridge, MA, USA

^5^Erasmus MC, University Medical Center Rotterdam, Department of Clinical Genetics, Rotterdam, the Netherlands

## Supplementary material

##
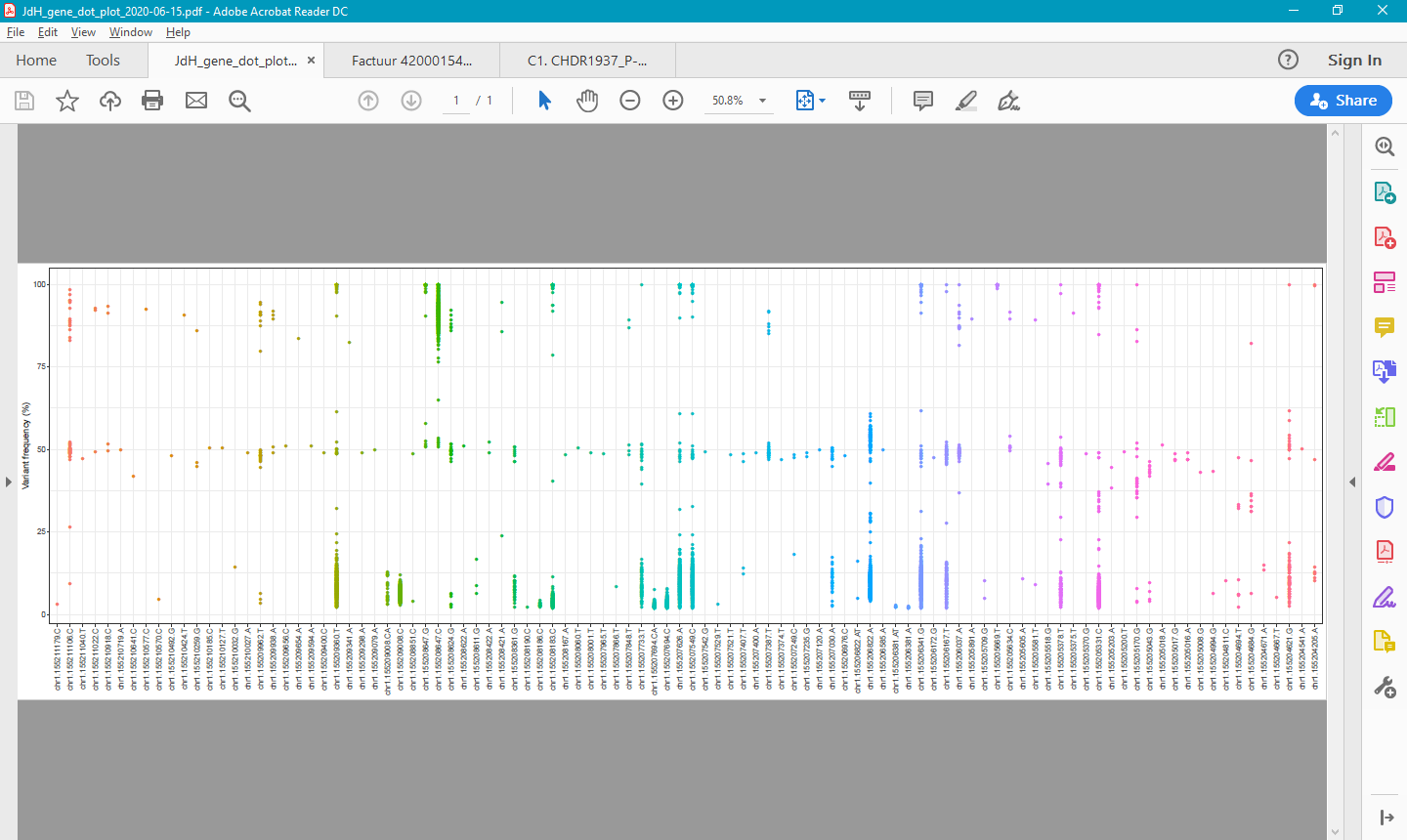


Supplementary figure 1 Dot plot of the variant frequencies using Q5 polymerase of all intronic and exonic variants in 1295 PD samples. Most samples had multiple intronic variants.

Supplementary figure 2 Intronic and exonic variants using Takara polymerase on the first 216 samples that had an exonic variant according to the initial screening. Some abnormal variant frequencies are still seen in high-repeat intronic regions, considered technical noise.

Supplementary figure 3 Intronic and exonic variants of all samples that were run with Takara polymerase. Some abnormal variant frequencies are still seen in high-repeat intronic regions, considered technical noise.
